# Narrative Preparedness: Policy-Makers Must Engage With People’s Values and Experiences to Ensure Effective Implementation of Interventions in Health Emergencies: Comment on "Health Preparedness and Narrative Rationality: A Call for Narrative Preparedness"

**DOI:** 10.34172/ijhpm.8627

**Published:** 2024-08-27

**Authors:** Catherine Grant

**Affiliations:** Institute of Development Studies, University of Sussex, Brighton, UK.

**Keywords:** Trust, Narrative Preparedness, Transdisciplinarity, Policy, Health

## Abstract

Engebretsen and Baker’s conceptual paper "*Health Preparedness and Narrative Rationality: A Call for Narrative Preparedness*" advocates for the adoption of narrative preparedness in addition to health preparedness, emphasising the importance of engaging with people’s stories and values during health emergencies. This ensures that policy-makers and health authorities gain the trust of communities as there is evidence this leads to improved outcomes. Their key argument is that science cannot be used effectively in policy unless it makes sense to people and is presented in a way that resonates with their values. This commentary draws on the wider literature and some key examples showing the wisdom of this approach. However, it also suggests that to be successful in integrating narrative preparedness in policy we need to look beyond working with health authorities and use a more transdisciplinary approach as well as addressing both the process and normative challenges in its adoption.

## Introduction: Narrative Preparedness – Understanding the Concept

 In the realm of public health, the importance of preparedness has been underscored time and again, particularly in the face of global health emergencies such as pandemics. However, while traditional health preparedness has focused largely on logistical and scientific aspects, Engebretsen and Baker argue for the necessity of narrative preparedness as a complementary approach. The paper uses Fischer’s narrative paradigm to argue that no matter how scientifically something is argued, it will always be a story, an interpretation of the world that is historically and culturally grounded and shaped by humanity.^1^ Narrative preparedness entails the capacity to engage with and comprehend the diverse narratives surrounding health crises, acknowledging the values embedded within them and their significance in shaping public perceptions and behaviours.

 This commentary elucidates the concept of narrative preparedness, explores its theoretical underpinnings, and discusses its implications for policy-makers and the public including the need to work with other disciplines in a transdisciplinary approach to ensure narrative preparedness is well integrated across the technical pillars of outbreak preparedness and response. This approach needs to align with the objectives and priorities of those technical pillars so these insights can help them to adapt, refine and contextualise their interventions. This requires buy-in from the technical leaders to collaborate and see the value in utilising social insights to adapt and improve their work, which is not always guaranteed (UK Public Health Rapid Support Team in Grant 2024).^2^ It also discusses both the normative and process barriers to implementing this approach.

## The Significance of Narrative Rationality

 Narrative preparedness expands upon the notion of health preparedness by emphasising the importance of narrative rationality alongside scientific rationality. In the context of public health emergencies such as the COVID-19 pandemic, narrative rationality plays a crucial role in shaping individuals’ perceptions and behaviours. This is based upon both structural and material coherence, how it coheres for people both internally and its external consistency (p. 5).^3^ Vaccine hesitancy and anti-vaccination narratives, for instance, are not solely driven by scientific evidence but are deeply rooted in cultural, social, and personal narratives. By examining these narratives through the lens of narrative rationality, policy-makers can gain insights into the underlying values and beliefs that influence public responses to health interventions. This approach calls for a deeper engagement with the stories and lived experiences of individuals and communities affected by health crises, recognising that these narratives often diverge from the rationality of science. Drawing upon Fisher’s narrative paradigm, which theorises that humans are inherently storytellers who interpret their experiences through narrative rationality, narrative preparedness advocates for a more holistic approach to public health communication and policy-making.

 An example from my geographical area of expertise of how narrative preparedness can be effective includes during the 2014-2016 West Africa Ebola outbreak. Fairhead outlines “social accommodations” that need to be made.^4^ During crises established norms of cooperation and coexistence can be violated, for example, one of the key transmission pathways of Ebola was unsafe burials, so they became medicalised and regulated, when previously they were handled within communities using important rituals. In Sierra Leone “*for many, mortuary practices are orchestrated to enable the dead person to accede to the ‘village of the ancestors’ where they reunite with the dead and live a very similar life to those on earth and continue to participate in affairs on earth*.”^5^ If these specific mortuary practices are not fulfilled the dead are condemned cause problems for those in the living world. This cultural importance meant that people resisted the response teams and attempted to adhere to traditional funerary practices, which exacerbated the epidemic and some internments became “super-spreading” events. Bans and fines for burying, sheltering or treating suspected Ebola patients and corpses were implemented by governments in Liberia, Guinea and Sierra Leone.^6^ However, traditional burials are important, as they are controlled by the female and male societies who are key to local and regional politics. Therefore alternative burial practices needed to work with communities to understand and accommodate cultural beliefs and needs surrounding burials, as such accommodations were more likely to be adhered to by local populations. As Richards wrote “*epidemiologically safe burial is unsafe from a social and spiritual perspective”* (p. 52).^7^ This marked a pivotal moment as epidemic response agencies started to recognise that comprehending local social dynamics and contexts can mitigate the extra costs and harm associated with “context-blind” interventions.^8^

## Trust: Characterological Coherence

 Engebretsen and Baker emphasise that it is important to create policies that reflect broader social, political, environmental and economic factors in society and this requires sustained “whole of society” efforts (p. 3).^3^ A key part of this approach is establishing trust, the person outlining the facts and policies need to be trusted and believed by the community (Characterological coherence in the authors’ model). The level of trust the population has in policy-makers and health authorities during an outbreak is influenced by historical and social contexts as well as policy decisions. This trust fluctuates based on past and present actions and realities, and Grant’s conceptual model expands on this as it emphasises the complexities of trust shaped by communities’ historical experiences with medicine, the effectiveness of health systems, social context, colonial legacies, public authority (dis)trust, and social determinants of health.^9^ As the world becomes increasingly interconnected and transdisciplinary, innovative approaches are essential to address these dynamics. A holistic, context-driven strategy that prioritises building trust with communities and tackles the new challenges arising from contemporary pandemics and epidemics is crucial for enhancing future preparedness efforts.^9^

 To illustrate this with a case example, Engebretsen and Baker highlight how this perspective applies to vaccine decision-making. They emphasise that individuals prioritise their lived experiences and the potential risks to their loved ones over a generalised risk assessment (p. 4).^3^ Vaccine uptake is a crucial component of pandemic response and future preparedness. In the COVID-19 era there was primarily a focus on supply issues, highlighting global injustices in vaccine distribution and emphasising the need for African countries to benefit from international deregulation and financing initiatives like COVAX. However, it is also important to consider that vaccine uptake and demand are perceived to be influenced by hesitancy, often linked to the global anti-vax movement and the spread of misinformation, exacerbated by a social media “infodemic” reaching African populations.

 These debates often overlook the socio-political contexts in which vaccine technologies are introduced and interpreted in Africa and the intersections between demand and supply. Trust in policy-makers is crucial in this context, as community trust ensures that policies resonate with local realities and encourages adherence to vaccination programs. Without trust, even the most well-intentioned policies can fail to achieve their desired outcomes.

 Leach and colleagues’ “vaccine anxieties” framework is a conceptual tool used to understand the complex and socially embedded reasons behind people’s desires for and concerns about vaccines. This framework considers the interplay of bodily, societal, and broader contextual factors that shape vaccine perceptions and behaviours. It highlights how trust dynamics, historical experiences with healthcare, socio-economic conditions, and cultural beliefs influence vaccine acceptance and hesitancy. By examining these multifaceted influences, the framework aims to provide a nuanced understanding of vaccine anxieties, which is crucial for developing effective public health strategies and improving vaccine preparedness.^10^

 This framework and Walter Fisher’s narrative paradigm lens offer valuable perspectives for understanding public responses to health interventions, particularly vaccines. While both approaches provide insights into how people interpret and respond to health-related information, they have distinct focuses and applications and Leach’s framework adds to, overlaps with, and potentially replaces elements of Fisher’s approach.

 Leach’s framework significantly expands upon Fisher’s narrative paradigm by emphasising socio-political and historical contexts, particularly in African settings. It considers how colonial legacies, political trust, and local experiences shape vaccine perceptions, providing a more nuanced understanding of the factors influencing public responses. Unlike Fisher’s approach, Leach’s framework explicitly considers both positive and negative anxieties, encompassing desires for vaccines as well as concerns about them. This dual nature of anxieties allows for a more comprehensive analysis of public attitudes towards vaccination.

 Another key addition in Leach’s framework is its exploration of the intersection between vaccine supply issues and geopolitical factors, and their influence on local perceptions and anxieties. This dimension is particularly relevant in the context of global health interventions and inequities in vaccine distribution. Furthermore, Leach’s framework incorporates culturally specific understandings of the body and health, adding a dimension not explicitly addressed in Fisher’s narrative approach.

 Despite these differences, both emphasise the importance of meaning-making, recognising how people construct understanding from their experiences and information. While Leach’s framework is more explicit about socio-political factors, both approaches acknowledge the significance of social context in shaping perceptions. Additionally, both frameworks move away from deficit models, rejecting the notion that public responses are simply due to a lack of information or understanding.

 Leach’s framework potentially replaces some elements of Fisher’s approach. By shifting focus from narratives to anxieties, it offers a more nuanced way to capture emotional and cognitive responses to vaccines. The framework’s specificity to vaccine issues makes it particularly suited for analysing responses in this context, potentially replacing more general narrative analysis. Moreover, Leach’s emphasis on the fluid and contingent nature of vaccine anxieties provides a more dynamic model than Fisher’s approach. It builds upon narrative approaches like Fisher’s by offering a more contextualised, dynamic, and vaccine-specific model for understanding public responses. It adds crucial dimensions particularly relevant to global health contexts, while retaining the emphasis on meaning-making and social context that is central to narrative approaches. This framework provides a valuable tool for researchers and policy-makers seeking to understand and address the complex factors influencing vaccine acceptance and hesitancy in diverse cultural and socio-political contexts.

 By examining the socially rooted factors influencing people’s attitudes towards COVID-19 vaccines and how these factors interact with vaccine supply, accessibility, and distribution dynamics in rapidly evolving epidemic scenarios, they emphasise the critical role of fostering and sustaining community trust in policy-makers. This approach is crucial for enhancing the efficacy of vaccination efforts and broader public health initiatives.

## Implications for Policy-Makers, the Public and Process and Normative Challenges

 For policy-makers, narrative preparedness provides a crucial framework for tackling health controversies and effectively conveying medical information. The key to successful health policy intervention lies not only in presenting the facts but in understanding how these facts resonate with people and why they matter to them (p. 7).^3^ By acknowledging and engaging with diverse narratives, policy-makers can design more inclusive and culturally sensitive public health interventions that resonate with different segments of society. Moreover, narrative preparedness fosters a deeper understanding of epistemic controversies related to health crises, enabling policy-makers to navigate complex issues with greater nuance and empathy. For the public, narrative preparedness underscores the importance of engaging with diverse narratives during health emergencies. By recognising the validity of individuals’ stories and lived experiences, the public can contribute to the development of more responsive and inclusive public health policies. Moreover, narrative preparedness empowers individuals to critically evaluate the narratives propagated by various stakeholders and make informed decisions about their health and well-being.

 The integration of transdisciplinarity with Fisher’s narrative preparedness framework presents a promising avenue for enhancing our understanding and management of complex health challenges. This approach necessitates a more comprehensive and nuanced incorporation of diverse perspectives from fields such as anthropology, sociology, psychology, and communication studies, alongside traditional public health and epidemiology. Rather than merely including social sciences broadly, this integration aims to create new, synthesised frameworks that transcend disciplinary boundaries, offering a more holistic approach to narrative preparedness.^11^

 The 2014-2016 Ebola outbreak in West Africa provides a compelling example of successful transdisciplinary integration. The inclusion of anthropologists such as Paul Richards and Sharon Abramowitz in preparedness and policy groups led to crucial insights into local burial practices and social structures.^7,12^ These contributions were essential for developing effective containment strategies that were culturally sensitive and ultimately more successful. This case demonstrates how transdisciplinary approaches can significantly enhance narrative preparedness by bridging the gap between scientific knowledge and local cultural contexts.^13^

 However, there are process and narrative challenges to implementing this approach. The process challenges, practical and logistical difficulties, include firstly the integration of narrative and health preparedness. Developing frameworks that effectively combine narrative preparedness with traditional health preparedness can be complex. This requires transdisciplinary approaches and collaboration between public health experts and social scientists. Secondly, establishing effective channels for engaging with communities to gather and understand their stories and values is challenging. This involves not only collecting narratives but also ensuring they are meaningfully incorporated into policy-making processes. Thirdly, authorities and policy-makers need training in narrative skills and cultural competence to effectively understand and integrate community stories into their work. This involves creating new educational programmes and resources. Finally, it is necessary to develop methods to evaluate the impact of narrative preparedness on health outcomes. This includes creating metrics and tools to measure how well narrative approaches influence trust, adherence to guidance, and overall health results.

 Normative challenges, difficulties related to values, ethics, beliefs, and cultural norms, involve aligning actions and policies with the values and expectations of stakeholders, which can include societal norms, ethical standards, and community beliefs. In the context of adopting narrative preparedness, normative challenges include ensuring that community narratives are valued. Firstly, there is a need to shift the traditional focus of health preparedness to include and value community stories and perspectives. This requires a cultural change within health institutions that have traditionally prioritised quantitative data over qualitative insights. Secondly, it is key to ensure that engagement with community narratives genuinely builds trust rather than being seen as tokenistic. This involves consistent, transparent, and respectful communication and actions that demonstrate the value placed on community input. Thirdly, policy-makers need to ensure that scientific guidance is aligned with the values and beliefs of the communities they serve. This requires a normative shift in how science is communicated, emphasising empathy and cultural relevance.

 Additionally, integrating expert knowledge with lay perspectives can create tensions. There needs to be a balance between respecting scientific evidence and acknowledging the lived experiences and values of the community, without compromising on health standards. For example, the integration of scientific expertise with local or lay knowledge often creates tensions that must be carefully navigated. For instance, during the 2018-2020 Ebola outbreak in the Democratic Republic of Congo, conflicts arose between biomedical approaches to containment and local understandings of the disease. Health workers faced resistance when attempting to implement isolation measures that conflicted with community care practices and mistrust of response teams was due to perceived inadequacies of the response effort, suspicion of mercenary motives, and violation of cultural burial norms.^14^ Similarly, in vaccine hesitancy contexts, scientific evidence supporting vaccine safety frequently clashes with personal narratives of perceived vaccine injuries. The challenge lies in acknowledging and addressing these narratives without undermining scientific consensus. Another issue is ensuring that the voices of marginalised and underrepresented groups are included and valued in narrative preparedness. This challenges existing power dynamics and requires deliberate efforts to include diverse perspectives. For example, traditional healing practices in many indigenous communities sometimes conflict with Western medical approaches, necessitating careful negotiation and mutual respect to integrate these knowledge systems effectively. Adapting policies to reflect community narratives and values may require significant changes to established practices and norms within public health and policy-making institutions. This can be met with resistance and require ongoing advocacy and support.

 To effectively integrate transdisciplinarity with narrative preparedness, several key strategies should be implemented. First, creating collaborative spaces where diverse experts, including social scientists, biomedical researchers, and community leaders, can co-develop frameworks for understanding and addressing health crises is crucial. Second, developing methodologies that give equal weight to scientific data and community narratives is essential, recognising that both contribute valuable insights to preparedness efforts. Third, training health practitioners in narrative competence will enable them to engage with and understand diverse storytelling traditions and their impact on health behaviours. Finally, establishing mechanisms for ongoing dialogue between scientific experts and community members is vital to build trust and facilitate knowledge exchange.

 By explicitly incorporating these transdisciplinary elements, the narrative preparedness framework can become more robust and effective in addressing complex health challenges. This approach not only enhances our understanding of diverse perspectives but also improves the efficacy of health interventions by ensuring they are culturally appropriate and locally accepted. As global health challenges continue to evolve, this integrated approach to narrative preparedness offers a promising path forward in bridging the gap between scientific expertise and community knowledge.

## Conclusion

 In conclusion, this commentary greatly appreciates the value of narrative preparedness and it represents a vital aspect of comprehensive health preparedness, particularly in the context of public health emergencies. By embracing narrative rationality and engaging with diverse narratives, policy-makers can foster trust, promote inclusivity, and enhance the effectiveness of public health interventions. This paper argues that moving forward, this approach will be most effective if narrative preparedness is integrated into transdisciplinary policy and practice as this will be essential for building resilient and responsive health systems capable of addressing the multifaceted challenges posed by future pandemics and other health emergencies. The paper highlights implications for policy-makers, urging them to recognise the value of narrative preparedness in addressing health controversies and fostering inclusive health preparedness for future pandemics, however this commentary also highlights the normative and process challenges to this.

## Ethical issues

 Not applicable.

## Conflict of interests

 Author declares that she has no competing interests.
